# Trends in UK meat consumption: analysis of data from years 1–11 (2008–09 to 2018–19) of the National Diet and Nutrition Survey rolling programme

**DOI:** 10.1016/S2542-5196(21)00228-X

**Published:** 2021-10-07

**Authors:** Cristina Stewart, Carmen Piernas, Brian Cook, Susan A Jebb

**Affiliations:** a Nuffield Department of Primary Care Health Sciences, University of Oxford, Oxford, UK

## Abstract

**Background:**

High meat consumption, particularly red meat and processed meat, negatively affects our health, while meat production is one of the largest contributors to global warming and environmental degradation. The aim of our study was to explore trends in meat consumption within the UK and the associated changes in environmental impact. We also aimed to identify any differences in intake associated with gender, ethnicity, socioeconomic status, and year of birth.

**Methods:**

Our study aimed to describe consumption of red, white, and processed meat within the UK, using data from the National Diet and Nutrition Survey rolling programme (2008–09 to 2018–19), and the associated changes in environmental impact. Meat consumption was based on disaggregated meat data, from 4 day food diaries that excluded all non-meat components of composite dishes. For each year surveyed, trends for meat intake were reported as mean grams per capita per day and linear-regression models were used to test for trends. We used multivariable linear-regression models to examine differences among consumers, as a percentage of food energy, by gender, ethnicity, equivalised household income, and year of birth.

**Findings:**

From 2008 to 2019, average meat consumption per capita per day decreased from 103·7 g (SE 2·3) to 86·3 g (2·9) per day (p_trend_<0·0001), including an absolute reduction in red-meat consumption of 13·7 g (p_trend_<0·0001), an absolute reduction in processed meat consumption of 7·0 g (p_trend_<0·0001), and a 3·2 g increase (p_trend_=0·0027) in white-meat consumption. Collectively, these changes were associated with a significant reduction in all six environmental indicators over the whole period. The two middle birth-year groups (people born in 1960–79 and 1980–99) and White individuals were the highest meat consumers. Meat intake increased over time among people born after 1999, was unchanged among Asian and Asian British populations, and decreased in all other population subgroups. We found no difference in intake with gender or household income.

**Interpretation:**

Despite the overall reduction in meat intake, reaching meat-consumption targets that align with sustainable diets will require a substantial acceleration of this trend.

**Funding:**

The Wellcome Trust, Our Planet Our Health programme (Livestock, Environment, and People).

## Introduction

Meat production is one of the largest contributors to global warming and environmental degradation. The livestock sector is responsible for about 15% of anthropogenic greenhouse-gas emissions (GHGE),^1^ while also driving deforestation, land degradation, and biodiversity loss.^2^ The UK Committee on Climate Change has called for a 20% reduction in consumption of beef, lamb, and dairy by 2050.^3^ Moreover, high consumption of animal products, particularly red meat and processed meat, also negatively affects human health.^4^ There is increasing evidence that high intakes of processed meat, and to a lesser extent red meat, lead to an increased risk of obesity,^5^ cardiovascular disease,^6^ and some forms of cancer.^4^ WHO has classified processed meat as a carcinogen and red meat as a probable carcinogen to humans.^7^ The relationship between red-meat and processed meat intake and colorectal cancer led the Scientific Advisory Committee on Nutrition (SACN) to recommend that adults in the UK with high intakes (>90 g/day) should reduce their intake to a maximum of 70 g per day.^8^

Reducing meat consumption could help protect the natural environment and improve human health. The EAT–*Lancet* Commission concluded that there needs to be a greater than 50% reduction in global red-meat consumption, among other dietary changes, to achieve a sustainable, healthy food system.^9^ In the UK, research groups have suggested that beef consumption needs to decrease by 89% to stay within planetary boundaries.^10^ Globally, the average per-capita consumption of meat, and the total amount of meat consumed, is increasing, driven by population growth, rising incomes, and sociocultural traditions that place a high value on eating meat.^11^, ^12^ However, large disparities in meat intake in different parts of the world remain; in some high-income countries such as the UK, per-capita consumption of meat is high but beginning to decline, while in many middle-income countries, such as China and those in east Asia, meat consumption is still rising.^11^ Moreover, within countries, attitudes towards meat consumption and meat reduction differ between subgroups of the population, as defined by age, gender, ethnicity, or income.^13^, ^14^

**Research in context**

**Evidence before this study**

The EAT–*Lancet* Commission concluded that global red-meat consumption needs to decrease by more than 50% to achieve a healthy sustainable diet. Globally, average per-capita consumption of meat is increasing, although there are large disparities in intake between different parts of the world. Moreover, attitudes towards meat consumption and meat reduction differ between subgroups of the population. To date, little is known about how much, and what types, of meat we are eating in the UK, how this consumption differs among population subgroups, and how it has changed over time.

**Added value of this study**

This study provides a detailed analysis of trends in meat intake, together with estimated associated changes in environmental impact, from a series of nationally representative surveys on food consumption within the UK. Our findings indicate that meat consumption is declining in the UK (–17·4 g per capita per day over a recent decade), with people consuming less red and processed meat but more white meat. We estimated that this was associated with a significant reduction across six different environmental indicators. People born in the 1980s and 1990s and White individuals were the highest meat consumers, and consumption has increased among people born after 1999.

**Implications of all the available evidence**

The results suggest that in the UK, self-reported red-meat and processed meat intake is decreasing slowly, although other sources of food-supply data are more equivocal and can even suggest an increase. There is consistent evidence, however, of an increase in white-meat consumption. There is a clear need for a greater focus on meat-eating habits if health and environmental targets are to be met. Differences in the trends in population subgroups suggest that a stratified approach to interventions might be required.

The aim of our study was to conduct an exploratory analysis of trends in meat consumption within the UK and the associated changes in environmental impact. We also aimed to identify any differences in intake associated with gender, ethnicity, and socioeconomic status. In particular, we aimed to explore trends for different years of birth. Understanding these trends could help tailor behavioural interventions and public health policies to population subgroups to accelerate progress towards health and environmental dietary targets.

## Methods

### Data source and sample

This study used data from years 1–11 (2008–09 to 2018–19) of the National Diet and Nutrition Survey (NDNS) rolling programme; a continuous, cross-sectional survey funded by Public Health England and the UK Food Standards Agency. The NDNS collects quantitative information regarding food consumption, nutrient intake, and nutritional status of the UK general population living in private households, with age and sex weighting to reflect population distributions.^15^, ^16^

Participants were drawn from postcode address files, in which the addresses were clustered into primary sampling units (PSUs) on the basis of postcode sectors and then randomly selected from across the UK. The methodology of the NDNS rolling programme has been described in detail elsewhere.^15^

### Dietary-data collection and processing

Dietary data were collected using consecutive, 4 day, estimated unweighed food diaries, with all days of the week equally represented. Meat consumption was based on disaggregated meat data that excluded all non-meat components of composite meat dishes and products.^16^

Trained coders and editors entered food-intake information into a modified version of the Medical Research Council Elsie Widdowson Laboratory dietary-assessment tool. For composite meals (eg, ready meals or homemade dishes), all ingredients were entered individually and allocated to the same recipe food group. A weight-change factor for the whole dish (from a similar recipe in McCance and Widdowson's Composite of Foods series) was applied to the raw weights recorded by the participant, to establish the weight of each cooked ingredient and the weight of the portion consumed. Detailed diary methodology and processing is provided in NDNS appendices.^16^

### Meat-grouping system and category definitions

We explored the consumption of total meat, red meat, white meat, processed meat, and fish, both as summary categories and as individual meat types, as reported in the NDNS-data files. All summary categories, except for processed meat, were used as defined by the NDNS. Red meat included beef, lamb, pork, and other red meat and offal (for the purpose of our analyses we grouped other red meat and offal together because they provided negligible contributions to red-meat intake). White meat included poultry and game birds, and fish included white fish, oily fish, canned tuna, and shellfish. We generated the summary category processed meat to include processed red meat, processed poultry, burgers, and sausages to match the International Agency for Research on Cancer's definition of processed meat. Results are presented for both summary categories and individual meat types; detailed descriptions of all categories are provided in the appendix (p 1).

### Sociodemographic information

Sociodemographic variables included self-reported age, gender, ethnicity, and equivalised household-income tertiles. Participants were categorised into four groups on the basis of year of birth (<1960, 1960–79, 1980–99, >1999), calculated by subtracting their age in years from the survey year in which they took part. Each survey year spanned 2 years, and for this calculation, we assumed participants completed the survey in the second year because date of birth and date of survey completion were not provided. The NDNS is a cross-sectional rolling programme and data analysed here span a decade. We focused on birth-year group, instead of age group, to reflect temporal trends (ie, an individual born in 1989 might behave differently to an individual of the same age born in 1978). Gender was categorised as men or women; ethnicity was categorised into four groups, that comprised White, Black or Black British, Asian or Asian British, and other ethnicity. We grouped any other group and mixed ethnic group together because there were too few people in these groups for meaningful analysis. Household income was equivalised to adjust for household size and composition.

### Environmental indicators

Environmental impact data for beef, lamb, pork, and poultry were obtained from Poore and Nemecek's Life Cycle Assessment (LCA) database,^17^ across six environmental indicators, including land use (in m^2^ per gram of meat), GHGE (in kilograms of CO_2_ equivalents produced per gram of meat), acidifying emissions (in grams of SO_2_ equivalents produced per gram of meat), eutrophying emissions (in grams of PO_4_m^3^^-^ equivalents per gram of meat), freshwater withdrawals (in litres per gram of meat), and stress-weighted water use (in litres per gram of meat). Land use includes multicropping, fallow phases, and economic allocation to crop coproducts. This is a global database that consolidates data on approximately 38 700 farms producing 40 different agricultural products in a meta-analysis comparing different food production systems from 570 studies. We used randomised and resampled mean data provided for each indicator per kilogram of retail weight for each meat type. For GHGE, we used 100 year factors with climate-carbon feedbacks from the Fifth Assessment Report of The Intergovernmental Panel on Climate Change. These data, per gram, were incorporated into our existing dataset and multiplied by per-capita intakes of each meat type per day to obtain environmental impacts for each indicator per day.

### Statistical analysis

Survey weights and PSUs were used to account for clustering in the sample and to attempt to reduce non-response bias to obtain nationally representative results. Full details of the weighting methodology can be found elsewhere.^15^

For each year surveyed, trends for meat intake were reported as mean grams per capita per day and percentage of consumers and mean grams per consumer per day. Consumers refers to people who reported consuming the item during the 4 day dietary recording period; non-consumers whose intake was 0 g for a particular meat were included in the per-capita analyses. We calculated the proportion of respondents self-identifying as vegetarian and vegan, and the proportion of adult consumers (≥19 years) meeting SACN recommendations of a maximum of 70 g per day of red-meat and processed-meat consumption. Test for trends were done using linear regression models adding survey year as a continuous term in the models.

We investigated overall trends in meat consumption over time and in population subgroups using a multivariate linear-regression model predicting meat intake in relation to time (survey year), with an interaction term between year and each predictor (gender, ethnicity, equivalised household-income tertiles, and birth-year group). For the subgroup analyses, meat intake was expressed as a percentage of food energy among people who consumed meat in the 4 day dietary recording period to alleviate the potential bias caused by differences in reported energy intake. We also calculated the proportion of non-consumers by birth-year group.

We reported daily per-capita trends of environmental emissions and uses across all six indicators for beef, pork, lamb, and poultry both individually and collectively, for each survey year. Linear-regression models were used to test for trends over time, with survey year added as a continuous term in the models.

All analyses were carried out using Stata IC version 14.1. p<0·05 was set to denote statistical significance.

### Role of the funding source

The funders of the study had no role in study design, data collection, data analysis, data interpretation, or writing of the report.

## Results

The sample population studied consisted of 15 655 individuals aged 1·5–96 years, of which 360 (2·3%) self-reported as being vegetarian or vegan (table 1).

**Table 1:** Demographic characteristics from the National Diet and Nutrition Survey rolling programme, 2008–09 to 2018–19

		**n (%)**
Number of observations		15 655
Gender		
	Men	7207 (46·0%)
	Women	8448 (54·0%)
Age		
	≤10 years	4386 (28·0%)
	11–17 years	2922 (18·7%)
	18–40 years	3028 (19·3%)
	41–59 years	2828 (18·1%)
	≥60 years	2491 (15·9%)
Birth-year group		
	<1960	3198 (20·4%)
	1960–79	3031 (19·4%)
	1980–99	3706 (23·7%)
	>1999	5720 (36·5%)
Ethnicity		
	White	14 026 (89·6%)
	Black or Black British	373 (2·4%)
	Asian or Asian British	721 (4·6%)
	Other	519 (3·3%)
	Missing	16 (0·1%)
Equivalised household-income tertiles		
	Lowest tertile	4601 (29·4%)
	Middle tertile	4407 (28·2%)
	Highest tertile	4614 (29·5%)
	Missing	2033 (13·0%)
Self-reported diet type		
	Vegetarian	334 (2·1%)
	Vegan	26 (0·2%)
	Neither vegetarian nor vegan	15 294 (97·7%)
	Do not know	1 (0·01%)

We found a 3% point reduction (p=0·0068) in the proportion of respondents who reported eating meat during the 4 day recording period (table 2) between 2008 and 2019. There was an 11% point reduction in the proportion of people consuming red meat (p<0·0001) and a 7% point reduction in the proportion of people consuming processed meat (p=0·0003) from 2008 to 2019, largely because of a lower proportion of people who consumed lamb (13% point reduction, p<0·0001), beef (9% point reduction, p=0·0004), and processed red meat (8% point reduction, p=0·0002). We recorded a 3% point increase (p=0·0055) in the proportion of respondents who self-identified as vegetarian or vegan over time, with 5% self-identifying as such in the most recent survey year (appendix p 2).

**Table 2:** Consumption per capita by meat category, 2008–09 to 2018–19

	**2008–09**	**2009–10**	**2010–11**	**2011–12**	**2012–13**	**2013–14**	**2014–15**	**2015–16**	**2016–17**	**2017–18**	**2018–19**	**p_trend_**
**Total meat**												
Grams per day (SE)	103·7 (2·3)	97·4 (3·3)	99·3 (2·56)	94·1 (2·2)	95·4 (2·3)	94·5 (2·3)	99·9 (3·0)	93·6 (2·5)	93·0 (3·1)	87·9 (2·4)	86·3 (2·9)	<0·0001
Percentage of consumers	96%	95%	95%	95%	97%	95%	95%	95%	93%	94%	93%	0·0068
**Total red meat**												
Grams per day (SE)	37·4 (1·4)	31·4 (1·3)	31·9 (1·4)	30·6 (1·3)	32·1 (1·5)	28·6 (1·4)	30·2 (1·5)	28·2 (1·2)	29·3 (1·7)	25·8 (1·2)	23·7 (1·3)	<0·0001
Percentage of consumers	80%	76%	74%	76%	79%	75%	74%	73%	72%	71%	69%	<0·0001
**Total white meat**												
Grams per day (SE)	32·5 (1·3)	33·4 (2·5)	35·1 (1·7)	32·8 (1·3)	32·7 (1·2)	33·2 (1·6)	39·6 (1·8)	38·7 (1·7)	38·2 (1·8)	36·2 (1·7)	35·7 (1·9)	0·0027
Percentage of consumers	76%	74%	75%	75%	77%	75%	78%	78%	78%	80%	79%	0·0020
**Total processed meat**												
Grams per day (SE)	33·8 (1·5)	32·6 (1·6)	32·3 (1·6)	30·7 (1·1)	30·6 (1·2)	32·6 (1·6)	30·1 (1·5)	26·7 (1·2)	25·5 (1·3)	25·9 (1·2)	26·8 (1·6)	<0·0001
Percentage of consumers	81%	80%	74%	79%	80%	79%	78%	74%	73%	76%	74%	0·0003
**Total fish**												
Grams per day (SE)	21·8 (1·2)	21·8 (1·0)	20·6 (1·0)	21·4 (1·1)	19·4 (1·1)	21·3 (1·4)	21·0 (1·5)	19·6 (1·0)	19·7 (1·0)	21·9 (1·5)	21·6 (1·2)	0·57
Percentage of consumers	63%	64%	61%	64%	61%	59%	63%	62%	62%	61%	63%	0·51
**Beef**												
Grams per day (SE)	19·0 (0·9)	18·6 (1·0)	18·2 (1·0)	17·0 (1·1)	17·2 (1·4)	14·9 (0·9)	16·8 (1·0)	15·7 (1·0)	16·7 (1·1)	14·5 (0·9)	13·3 (0·9)	<0·0001
Percentage of consumers	62%	62%	59%	60%	59%	59%	58%	57%	57%	57%	53%	0·0004
**Lamb**												
Grams per day (SE)	7·2 (0·7)	5·1 (0·6)	6·4 (1·0)	4·7 (0·7)	5·8 (0·7)	5·5 (0·7)	5·0 (0·8)	4·3 (0·6)	5·2 (0·8)	4·6 (0·6)	3·3 (0·5)	0·0002
Percentage of consumers	23%	18%	16%	15%	18%	16%	16%	16%	17%	16%	10%	<0·0001
**Pork**												
Grams per day (SE)	8·4 (0·7)	5·8 (0·6)	5·4 (0·6)	7·6 (0·7)	7·6 (0·8)	6·2 (0·7)	6·9 (0·7)	6·7 (0·7)	6·5 (0·8)	5·6 (0·6)	5·8 (0·6)	0·080
Percentage of consumers	27%	22%	17%	27%	27%	24%	26%	26%	24%	24%	24%	0·48
**Other red meat and offal**												
Grams per day (SE)	2·8 (0·5)	1·9 (0·3)	2·0 (0·3)	1·2 (0·2)	1·5 (0·2)	2·0 (0·3)	1·4 (0·3)	1·4 (0·2)	1·0 (0·3)	1·1 (0·2)	1·4 (0·3)	0·0002
Percentage of consumers	16%	16%	12%	13%	12%	14%	11%	11%	9%	9%	10%	<0·0001
**Poultry**												
Grams per day (SE)	32·0 (1·3)	32·8 (2·5)	34·8 (1·7)	32·4 (1·3)	32·1 (1·2)	32·4 (1·6)	38·9 (1·8)	38·3 (1·7)	37·7 (1·9)	35·8 (1·7)	35·3 (1·9)	0·0024
Percentage of consumers	76%	73%	75%	75%	77%	74%	78%	77%	78%	79%	79%	0·0021
**Game birds**												
Grams per day (SE)	0·6 (0·1)	0·6 (0·1)	0·3 (0·1)	0·4 (0·1)	0·6 (0·2)	0·9 (0·5)	0·7 (0·3)	0·4 (0·1)	0·5 (0·1)	0·4 (0·1)	0·4 (0·1)	0·72
Percentage of consumers	3%	3%	1%	3%	3%	3%	2%	3%	3%	2%	2%	0·90
**Processed red meat**												
Grams per day (SE)	16·2 (0·8)	17·0 (1·3)	16·2 (0·8)	15·9 (0·7)	15·4 (0·8)	17·4 (1·0)	15·2 (0·8)	14·7 (0·7)	13·9 (0·9)	14·2 (0·8)	13·3 (0·8)	0·0005
Percentage of consumers	70%	70%	66%	70%	68%	70%	65%	63%	63%	66%	62%	0·0002
**Burgers**												
Grams per day (SE)	2·9 (0·4)	3·2 (0·4)	3·0 (0·4)	2·8 (0·3)	3·2 (0·4)	3·3 (0·4)	3·2 (0·4)	2·1 (0·3)	2·2 (0·3)	2·0 (0·3)	3·1 (0·5)	0·075
Percentage of consumers	14%	13%	12%	12%	14%	13%	14%	10%	10%	11%	12%	0·035
**Sausages**												
Grams per day (SE)	14·6 (1·0)	12·3 (0·7)	13·1 (1·0)	12·1 (0·8)	12·0 (0·8)	11·9 (0·8)	11·6 (0·9)	9·9 (0·6)	9·4 (0·7)	9·7 (0·7)	10·4 (0·8)	<0·0001
Percentage of consumers	44%	40%	37%	39%	41%	41%	41%	37%	36%	38%	39%	0·060
**Processed poultry**												
Grams per day (SE)	0	0·1 (0)	0	0	0·1 (0·0)	0	0	0	0	0	0	0·0039
Percentage of consumers	0%	0%	0%	0%	0%	0%	0%	0%	0%	0%	0%	0·032
**White fish**												
Grams per day (SE)	8·7 (0·7)	7·9 (0·5)	9·2 (0·6)	7·9 (0·5)	7·8 (0·7)	8·1 (0·9)	7·6 (0·6)	7·2 (0·5)	6·5 (0·5)	7·7 (0·9)	7·9 (0·8)	0·033
Percentage of consumers	35%	33%	35%	33%	31%	31%	32%	32%	31%	29%	31%	0·014
**Oily fish**												
Grams per day (SE)	7·4 (0·7)	7·8 (0·7)	6·2 (0·7)	7·6 (0·7)	6·8 (0·8)	8·2 (0·9)	8·0 (1·1)	6·9 (0·7)	7·6 (0·6)	8·2 (0·9)	7·1 (0·7)	0·56
Percentage of consumers	24%	24%	23%	24%	25%	24%	27%	27%	27%	28%	27%	0·016
**Canned tuna**												
Grams per day (SE)	3·3 (0·4)	3·4 (0·3)	3·0 (0·4)	3·4 (0·4)	2·8 (0·3)	2·7 (0·3)	3·3 (0·5)	3·4 (0·3)	3·0 (0·4)	3·9 (0·6)	4·0 (0·5)	0·24
Percentage of consumers	17%	22%	19%	20%	20%	16%	18%	20%	18%	21%	22%	0·38
**Shellfish**												
Grams per day (SE)	2·4 (0·3)	2·7 (0·4)	2·2 (0·4)	2·6 (0·4)	1·9 (0·3)	2·3 (0·3)	2·1 (0·4)	2·2 (0·3)	2·6 (0·4)	2·1 (0·4)	2·6 (0·3)	0·68
Percentage of consumers	15%	17%	12%	15%	13%	15%	15%	16%	16%	15%	18%	0·22

N=15 655. Data from the National Diet and Nutrition Survey rolling programme.

**Figure 1:**
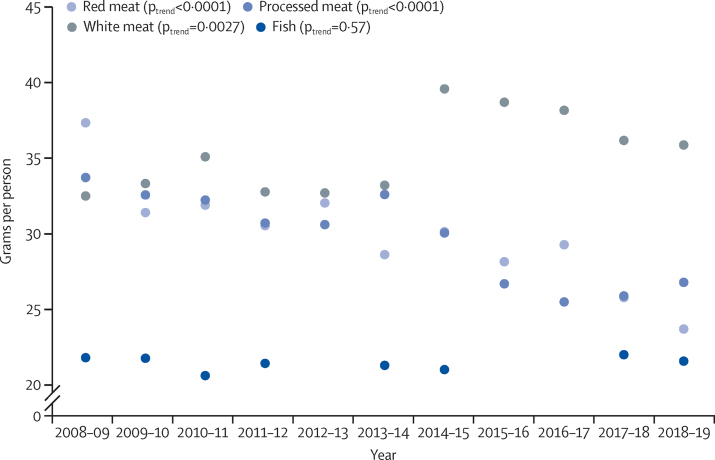
Consumption per capita per day by meat categories, 2008–09 to 2018–19 N=15 655. Data from the National Diet and Nutrition Survey rolling programme.

**Figure 2:**
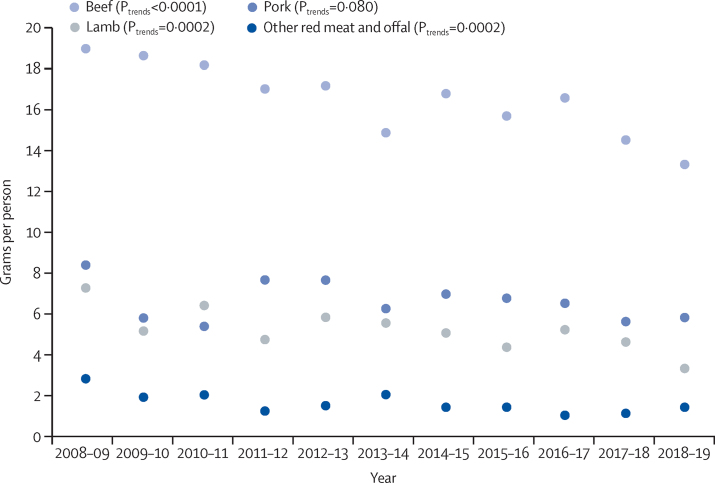
Consumption per capita per day by red-meat types, 2008–09 to 2018–19 N=15 655. Data from the National Diet and Nutrition Survey rolling programme.

Average daily meat intake per capita decreased from 103·7 g (SE 2·3) to 86·3 g (2·9), a reduction of 17·4 g (p_trend_<0·0001; table 2). This change included a 13·7 g reduction in red-meat consumption (37·4 g, SE 1·4, to 23·7 g, 1·3; p_trend_<0·0001), a 7·0 g reduction in processed meat consumption (33·8 g, SE 1·5, to 26·8 g, 1·6; p_trend_<0·0001), and a 3·2 g increase in white-meat consumption (32·5 g, SE 1·3, to 35·7 g, 1·9; P_trend_=0·0027; figure 1). There was no significant change in per-capita consumption of fish (–0·2 g per day, p_trend_=0·57). We noted a reduction in consumption of all individual red-meat and processed meat types except for pork and burgers (table 2). For red meat, the reduction was greatest for beef (–5·7 g; p_trend_<0·0001) and lamb (–3·9 g; p_trend_=0·0002; figure 2). Sausages were the largest contributor to the reduction in processed meat consumption (–4·2 g; p_trend_<0·0001). Trends in intake among people who reported eating meat during the recording period were similar, decreasing from 107·5 g (SE 2·2) to 92·3 g (2·9) per day, a reduction of 15·2 g (p_trend_<0·0001; appendix p 3).

The proportion of adult consumers meeting the SACN recommendation to limit red-meat and processed meat consumption to 70 g a day^8^ increased from 47% to 66% between 2008–09 and 2018–19 (p_trend_<0·0001). In the most recent survey, 74% of women met this recommendation compared with 57% of men (p<0·0001; appendix p 7).

**Figure 3:**
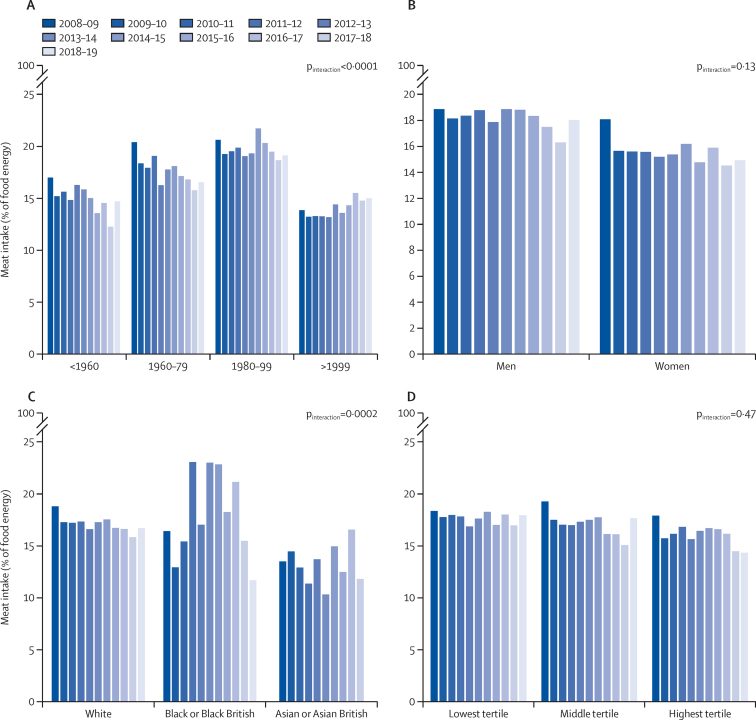
Differences in trends in meat intake among consumers, 2008–09 to 2018–19 N=15 015. Data from the National Diet and Nutrition Survey rolling programme. Differences in meat intake among consumers are analysed by birth-year group (A), gender (B), ethnicity (C), and equivalised household income (D). Estimates from multivariate linear-regression model predicting meat intake in relation to time (survey year), with an interaction term between year and each predictor.

Individuals born in the 1980s and 1990s consumed the most meat as a percentage of food energy (p<0·0001). The youngest (born after 1999) and oldest (born before 1960) groups were the lowest consumers (appendix p 4). With the exception of the youngest group, in which meat consumption has increased over time, intake in all other groups has decreased over the time period (p_interaction_<0·0001; figure 3A). Respondents born in the 1960s and 1970s had the highest proportion of non-consumers (6%; p=0·0024).

We did not record a significant difference in meat intake between men and women as a percentage of food energy (appendix p 4), and both men and women have decreased their consumption over time (figure 3B). Asian and Asian British individuals consumed the least meat (p=0·012; appendix p 4), although the analysis of trends in these groups and Black and Black British groups is limited by low sample size. Meat intake decreased over time among the White population (figure 3C). There was no significant difference in meat intake with household income (appendix p 4; figure 3D).

**Figure 4:**
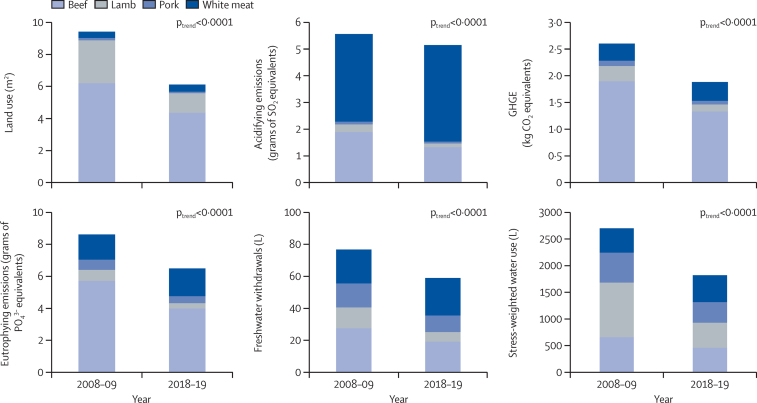
Daily environmental emissions/uses per capita across six indicators by meat type, 2008–09 to 2018–19 Data from the National Diet and Nutrition Survey rolling programme. Estimates taken from linear-regression models predicting environmental impact in relation to time (survey year). GHGE=greenhouse-gas emissions.

Overall changes in meat intake were estimated to be associated with a significant reduction in all six environmental indicators associated with meat production, including land use (–35%; p<0·0001), GHGE (–28%; p<0·0001), acidifying emissions (–21%; p<0·0001), eutrophying emissions (–25%; p<0·001), freshwater withdrawals (–23%; p<0·0001), and stress-weighted water use (–33%; p<0·0001; figure 4; appendix pp 5–6).

## Discussion

In the UK between 2008–09 and 2018–19, average daily meat consumption decreased by approximately 17·4 g per capita per day, with individuals consuming less red and processed meat, more white meat, and the same amount of fish. The proportion of meat consumers decreased by 3% points whereas the proportion of individuals identifying as vegetarian or vegan increased by 3% points. Intake was highest in the White population, but decreased over time, with no evidence of trends in minority ethnic groups. Individuals born in the 1980s and 1990s ate more meat than those born in other decades, and younger people have increased their intake over time, by contrast with people born before 1999. The observed changes in meat consumption were estimated to be associated with a significant reduction across all six environmental indicators.

A major strength of the present study is that it uses contemporary data from the NDNS, the only survey that captures nationally representative data on food consumption within the UK, including estimates of meat intake from disaggregated composite dishes. This detailed information allowed for changes in consumption to be expressed in subtle shifts in the type of foods or composite dishes that are consumed. Moreover, linking to data across six important environmental indicators allows us to explore how the environmental effects associated with meat consumption have been attenuated by the changes observed. Under-reporting of intake is an inherent limitation to self-reported dietary-assessment methods.^18^ However, expressing differences in intake as a percentage of food energy can help to reduce bias, particularly when comparing consumption across population subgroups. There are also limitations in the quality and availability of environmental impact data, given the intricacy and complexity of the food system. Because of the constraints of the LCA database, we were not able to include fish in the environmental analyses. However, as fish consumption has not changed over the past decade, it is unlikely to have influenced this outcome. Further, the estimated changes in environmental impact reported here reflect the potential changes associated with meat intake alone. These changes will be partly offset by the environmental impact of foods consumed in place of meat products. However previous research has shown that the environmental impact of meat production is higher than other food categories.^19^ Moreover, the LCA data used in our study are based on averages from global food-production systems; therefore the estimates we provide in relation to the UK consumption are approximates. However, our focus on trends over time rather than absolute intakes mitigates the effects of these limitations on the overall findings.

Consistent with observations in other countries, our results show a shift from red-meat towards white-meat consumption.^14^, ^20^ By contrast with other studies, however, we did not find any significant difference in meat intake between men and women (when expressed as a proportion of energy intake),^21^, ^22^, ^23^ or with household income, a marker of socioeconomic position. Previous studies in the UK have suggested that a socioeconomic gradient exists with meat consumption; however, this research has focused on red-meat and processed meat intake only.^24^, ^25^ Our focus on total meat, and not individual meat types, might explain this discrepancy, because it has been suggested previously that lower-income households consume more red meat and processed meat and less white meat.^26^ Drawing any firm conclusions on trends in meat intake in Asian, Asian British, Black, and Black British populations is difficult given the small sample size and the observed fluctuations over time that create considerable uncertainty in the trend.

Most previous studies have explored differences in intake with age groups, rather than birth-year groups. A trend analysis of the National Health and Nutrition Examination Survey in the USA reported that both the youngest (20–34 years) and oldest (≥65 years) age groups had increased their meat consumption over time, whereas the two middle-age groups had decreased their consumption.^27^ Our observation that the youngest (born after 1999) and the oldest (born before 1960) groups consumed the lowest amounts of meat sheds light on differences in behaviour between birth cohorts. Generation Z (born after 1999) were the lowest meat consumers in the first 7 years of the survey, but were the only group to report a higher intake over time. Millenials (born between 1980 and 1999) were consistently the highest meat consumers, with only a small reduction in reported intake observed over the surveyed period, whereas Boomers (born before 1960) were consistently low-level consumers with declining intakes reported over time. This result was unexpected given that a YouGov survey commissioned by The Eating Better Alliance found that 63% of those aged 11–18 years considered that the environment and climate change was one of the most important issues for the country. The survey also found that 29% of meat eaters aged 11–18 years said they wanted to reduce their meat consumption, and 25% of people aged 18 years already identified as vegetarian or vegan.^28^ However, respondents in the youngest group were aged 19 years or younger, and their eating habits as children are likely to be more reflective of their household than personal choice.

Our finding that meat consumption within the UK decreased between 2008 and 2019 aligns with meat-purchase data from the Family Food Survey of the Department for Environment, Food, and Rural Affairs. These data demonstrate a reduction in weekly purchases of meat at home (–37 g) but a small rise in meat purchases outside the home (8 g) per person, over the same period.^29^ Data obtained upon request from the Agriculture and Horticulture Development Board indicates that meat available for consumption in the UK has increased by 2·9 g per person per day (after adjustment for changes in population size).^30^ Although agricultural food-supply data can be useful to assess trends over time, it is not an accurate estimate of consumption, because food losses and waste can occur along the food chain. However, the small decrease reported in the NDNS and Family Food Surveys and the inherent uncertainty in these estimates, together with the apparent increase in meat available for consumption, precludes a firm conclusion that meat intake is convincingly in decline in the UK.

The UK Committee on Climate Change has set a target for at least a 20% reduction in beef, lamb, and dairy consumption by 2050.^3^ This reduction should be achievable, given that we observed a secular decrease of 30% in beef consumption and 55% in lamb consumption, from 2008 to 2019. However, this proposed reduction is modest and beef consumption in the UK has been estimated to need to decrease by 89% to stay within planetary boundaries.^10^ Such a reduction would be a substantial change in dietary habits, probably requiring substantial intervention. Indeed, we reported that 34% of individuals in the most recent survey year were exceeding the SACN recommendation of limiting red and processed meat intake to 70 g per day. Although the reductions in meat consumption and the associated environmental impacts observed here are positive, this trend will need to be accelerated. Understanding the intake patterns and trends in population subgroups indicates potential areas for future intervention that will be useful to researchers and public health professionals aiming to reduce meat intake to meet dietary targets for health and the environment. Clear evidence of the growing preference for poultry, which perhaps reflects dietary guidance for good health, suggests the need to raise awareness regarding the environmental impact of white meat.

In conclusion, dietary survey data suggest that meat consumption in the UK has decreased between 2008–09 and 2018–19, with individuals consuming less red and processed meat and consuming more white meat. The proportion of meat consumers has decreased over time, whereas the proportion of individuals identifying as vegetarian and vegan has increased. Understanding meat-consumption trends within subgroups of the UK population could help to tailor public health policies and behavioural interventions to accelerate the reduction in meat consumption to meet dietary targets for health and the environment.

## Data sharing

Data from the National Diet and Nutrition Survey years 1–11 (2008–09 to 2018–19) can be accessed on the UK Data Service (https://ukdataservice.ac.uk/). The datasets used and analysed in the present study are available from the corresponding author on reasonable request.

## Declaration of interests

We declare no competing interests.
