# Supplementary material for: Trends in UK meat consumption: analysis of data from years 1–11 (2008–09 to 2018–19) of the National Diet and Nutrition Survey rolling programme
Source: Lancet Planet Health. 2021 Oct 7;5(10):e699–708. doi: 10.1016/S2542-5196(21)00228-X (PMC8515514; doi:10.1016/S2542-5196(21)00228-X)
Supplement: Supplementary appendix [file mmc1.pdf]

THE LANCET
Planetary Health

Supplementary appendix

This appendix formed part of the original submission and has been peer reviewed. We post it as supplied by the authors.

Supplement to: Stewart C, Piernas C, Cook B, Jebb SA. Trends in UK meat consumption: analysis of data from years 1–11 (2008–09 to 2018–19) of the National Diet and Nutrition Survey rolling programme. *Lancet Planet Health* 2021; **5:** e699–708.

Appendix – Trends in UK Meat Consumption: Analysis of the National Diet and Nutrition Survey Rolling Programme Years 1-11 (2008/09-2018/19)

Table 1. Descriptions of individual meat and fish types and summary categories

Meat/Fish Item	Description
Beef	Any muscle meat from beef or veal
Lamb	Any muscle meat from mutton or lamb
Pork	Any muscle meat from pork (not bacon or ham)
Processed red meat	Manufactured, cured and/or dried meat, including bacon and ham
Other red meat	Any muscle meat from goat or venison
Burgers	Any meat consumed as a burger (not poultry). Grill steaks
Sausages	Any meat consumed as a sausage
Offal	Brain, heart, kidney, tongue, tripe, liver, oxtail, liver pate
Poultry (white meat)	Any muscle meat from chicken or turkey
Processed poultry	Manufactured, cured and/or dried meat, including chicken paste
Game birds	Any muscle meat from duck, goose, partridge, pheasant
White fish	Ayr, catfish, caviar, cod, cod roe, coley, dover sole, flounder, haddock, hake, halibut, hoki, john dory, lemonsole, ling, marlin, monkfish, mullet (red and grey), sake, plaice, pollack, red fish, red snapper, rock salmon/dogfish, rohu, sea bass, sea bream, shark, tilapia, turbot, whiting
Oily fish	Anchovies, bloater, carp trout, eel mackerel, herring, hilsa, kipper, jack fish, pangas, pilchards, salmon (including canned), sardines, sprats, swordfish, tuna (fresh only), whitebait
Canned tuna	Tuna (canned only)
Shellfish	Abalone, clams, cockles, crab, lobster, mussels, octopus, oysters, prawns, scallops, shrimps, squid, whelks, winkles
Total fish	White fish, oily fish, canned tuna and shellfish
Total meat	Total red meat, total white meat and total processed meat
Total red meat	Beef, lamb, pork, other red meat and offal
Total white meat	Poultry and game birds
Total processed meat	Processed red meat, burgers, sausages and processed poultry

All descriptions, with the exception of 'total processed meat', were taken from the National Diet and Nutrition Survey data files.

Table 2. Proportion of respondents who self-identified as vegetarian or vegan within the NDNS Rolling Programme (2008/09-2018/19).

Survey Year	Proportion of respondents N (%)
2008/09	29 (1·9%)
2009/10	22 (2·5%)
2010/11	26 (1·4%)
2011/12	52 (3·4%)
2012/13	31 (2·5%)
2013/14	29 (2·5%)
2014/15	32 (2·7%)
2015/16	35 (3·2%)
2016/17	31 (3·7%)
2017/18	34 (3·9%)
2018/19	33 (4·5%)

Abbreviations: NDNS, National Diet and Nutrition Survey

Table 3. Per consumer trends (g/day (SE)) by meat category in NDNS Rolling Programme (2008/09-2018/19)

	2008/09	2009/10	2010/11	2011/12	2012/13	2013/14	2014/15	2015/16	2016/17	2017/18	2018/19	P trend
Total meat	107·5 (2·2)	102·1 (3·4)	104·2 (2·5)	99·4 (2·1)	98·9 (2·3)	99·0 (2·2)	104·7 (2·9)	98·6 (2·2)	100·0 (2·8)	93·7 (2·4)	92·3 (2·9)	<0·001
Total red meat	46·5 (1·6)	41·4 (1·5)	43·1 (1·7)	40·1 (1·4)	40·8 (1·7)	38·0 (1·5)	41·0 (1·7)	38·5 (1·3)	40·8 (1·8)	36·4 (1·5)	34·7 (1·5)	<0·001
Total white meat	42·7 (1·5)	45·2 (3·2)	46·7 (1·9)	43·6 (1·4)	42·5 (1·3)	44·1 (1·7)	50·8 (2·1)	49·9 (1·8)	48·7 (2·10)	45·1 (1·8)	45·7 (2·0)	0·052
Total processed meat	41·6 (1·5)	40·7 (1·8)	43·3 (1·8)	38·9 (1·2)	38·4 (1·3)	41·4 (1·7)	38·7 (1·7)	35·9 (1·1)	35·0 (1·3)	34·1 (1·4)	36·1 (1·9)	<0·001
Total fish	34·6 (1·5)	33·9 (1·3)	33·8 (1·7)	33·7 (1·6)	31·7 (1·4)	36·1 (1·8)	33·5 (2·1)	31·5 (1·4)	31·6 (1·3)	35·8 (1·9)	34·4 (1·5)	0·789
Beef	30·7 (1·2)	29·8 (1·3)	30·8 (1·3)	28·3 (1·4)	29·2 (1·8)	25·0 (1·1)	29·1 (1·3)	27·6 (1·2)	29·3 (1·5)	25·5 (1·2)	25·0 (1·4)	<0·001
Lamb	32·0 (1·8)	28·4 (1·7)	40·3 (3·4)	32·4 (2·9)	32·2 (1·9)	34·3 (2·4)	32·4 (3·5)	26·6 (2·0)	31·0 (2·5)	29·5 (2·4)	32·3 (3·0)	0·226
Pork	31·0 (1·7)	26·0 (2·1)	31·7 (2·4)	28·3 (1·6)	28·5 (2·0)	26·0 (2·2)	26·5 (1·6)	25·8 (2·1)	27·3 (2·4)	22·9 (1·7)	24·5 (1·9)	0·002
Other red meat & offal	17·1 (2·2)	12·0 (1·5)	16·5 (1·7)	9·1 (1·3)	12·3 (1·1)	14·1 (1·9)	13·2 (2·0)	13·1 (21·7)	11·0 (2·7)	12·4 (2·3)	13·7 (2·2)	0·221
Poultry	42·1 (1·5)	45·0 (3·2)	46·6 (1·9)	43·2 (1·3)	41·9 (1·3)	43·6 (1·7)	49·9 (2·0)	49·6 (1·8)	48·4 (2·1)	45·1 (1·8)	44·9 (2·1)	0·046
Game birds	18·1 (3·4)	21·3 (3·3)	22·0 (4·2)	17·1 (2·6)	23·0 (3·8)	31·2 (13·1)	31·2 (8·4)	14·8 (3·1)	16·6 (2·5)	17·4 (2·6)	18·3 (2·7)	0·686
Processed red meat	23·3 (1·1)	24·3 (1·6)	24·5 (1·1)	22·9 (0·9)	22·5 (1·0)	24·8 (1·2)	23·5 (1·0)	23·2 (0·9)	22·1 (1·0)	21·4 (1·0)	21·2 (1·0)	0·042
Burgers	21·1 (1·7)	23·7 (1·2)	24·6 (1·6)	23·1 (1·2)	22·6 (1·3)	25·7 (1·6)	22·8 (1·1)	21·9 (1·4)	22·3 (1·6)	18·8 (1·5)	25·6 (2·4)	0·924
Sausages	33·5 (1·6)	30·9 (1·4)	35·2 (1·5)	30·5 (1·2)	29·6 (1·1)	29·3 (1·4)	28·2 (1·5)	26·4 (1·0)	26·1 (1·3)	25·4 (1·2)	26·7 (1·5)	<0·001
Processed poultry	7·9 (1·2)	9·4 (2·9)	6·3 (0·9)	4·7 (0·9)	17·3 (3·5)	5·3 (0·7)	2·6 (1·1)	8·9 (2·3)	3·1 (0·6)	6·7 (1·8)	3·4 (0·3)	0·094
White fish	24·9 (1·3)	24·0 (0·9)	26·4 (1·4)	24·1 (1·2)	25·1 (1·3)	25·8 (1·9)	23·7 (1·4)	22·5 (1·1)	21·2 (1·0)	26·3 (2·4)	25·0 (1·7)	0·455
Oily fish	30·9 (1·8)	31·7 (1·8)	26·3 (1·7)	31·9 (1·9)	27·7 (1·8)	33·4 (2·5)	29·6 (3·4)	25·7 (1·5)	27·6 (1·5)	29·6 (2·0)	26·6 (1·9)	0·061
Canned tuna	19·4 (2·0)	15·7 (0·8)	15·4 (2·0)	17·4 (1·0)	14·2 (1·4)	16·7 (1·5)	17·9 (1·9)	16·6 (1·1)	17·4 (1·6)	18·8 (1·7)	18·3 (1·6)	0·391
Shellfish	16·5 (1·5)	15·3 (1·5)	18·5 (2·4)	17·4 (2·0)	14·7 (1·3)	15·0 (1·4)	14·3 (1·8)	13·9 (1·4)	16·20 (1·9)	14·4 (2·0)	14·2 (1·2)	0·103

Abbreviations: NDNS, National Diet and Nutrition Survey; SE, Standard Error

Table 4. Per consumer meat intake differences (% of food energy) with demographic groups in NDNS Rolling Programme (2008/09-2018/19)

	Beta	95% CI		P Value	P Interaction*
**Gender**	-0·76	-2·01	0·49	0·232	0·131
**Birth Cohort**					<0·001
1960-1979	3·40	1·56	5·23	<0·001	
1980-1999	3·63	1·67	5·59	<0·001	
>1999	-3·14	-4·52	-1·76	<0·001	
**Ethnicity**					<0·001
Black or Black British	-2·37	-10·24	5·50	0·554	
Asian or Asian British	-5·30	-9·42	-1·18	0·012	
Other	-1·76	-4·95	1·43	0·279	
**Eqv household income tertiles**					0·470
Middle tertile	0·91	-0·73	2·55	0·275	
Highest tertile	-0·46	-2·02	1·10	0·566	

Abbreviations: NDNS, National Diet and Nutrition Survey; Eqv, Equivalised; CI, confidence interval
Estimates from multivariate linear regression model. Each coefficient shows the difference in meat intake as a percentage of food energy compared with the reference category while adjusting for all other predictors of meat intake.
Reference categories are: men, birth cohort <1960, white ethnicity, equivalised household income lowest tertile.
*P joint interaction of each covariate with year, Wald test, P<0·05 to denote statistical significance.

Table 5. Daily environmental emissions/uses per capita by meat type in NDNS Rolling Programme (2008/09-2018/19)

	2008/09	2009/10	2010/11	2011/12	2012/13	2013/14
**Land Use (m2/g)**						
Beef	6·2 (0·29)	6·1 (0·33)	5·9 (0·32)	5·5 (0·35)	5·6 (0·45)	4·8 (0·29)
Lamb	2·7 (0·26)	1·9 (0·22)	2·4 (0·36)	1·7 (0·27)	2·1 (0·25)	2·0 (0·26)
Pork	0·1 (0·01)	0·1 (0·01)	0·1 (0·01)	0·1 (0·01)	0·1 (0·01)	0·1 (0·01)
Poultry	0·4 (0·02)	0·4 (0·03)	0·4 (0·02)	0·4 (0·02)	0·4 (0·01)	0·4 (0·02)
Total	9·4 (0·33)	8·5 (0·41)	8·8 (0·44)	7·8 (0·41)	8·3 (0·47)	7·4 (0·35)
**GHG emissions (kg CO2eq/g)**						
Beef	1·9 (0·09)	1·9 (0·10)	1·8 (0·10)	1·7 (0·11)	1·7 (0·14)	1·5 (0·09)
Lamb	0·3 (0·03)	0·2 (0·03)	0·3 (0·04)	0·2 (0·03)	0·2 (0·03)	0·2 (0·03)
Pork	0·1 (0·01)	0·1 (0·01)	0·1 (0·01)	0·1 (0·01)	0·1 (0·01)	0·1 (0·01)
Poultry	0·3 (0·01)	0·3 (0·02)	0·3 (0·02)	0·3 (0·01)	0·3 (0·01)	0·3 (0·02)
Total	2·6 (0·09)	2·5 (0·11)	2·5 (0·10)	2·3 (0·11)	2·3 (0·13)	2·1 (0·09)
**Acidifying Emissions (g SO2eq/g)**						
Beef	6·0 (0·28)	5·9 (0·33)	5·8 (0·31)	5·4 (0·34)	5·5 (0·44)	4·7 (0·28)
Lamb	1·0 (0·10)	0·7 (0·08)	0·9 (0·13)	0·7 (0·10)	0·8 (0·10)	0·8 (0·10)
Pork	1·2 (0·10)	0·8 (0·09)	0·8 (0·08)	1·1 (0·10)	1·1 (0·12)	0·9 (0·11)
Poultry	3·3 (0·13)	3·4 (0·25)	3·6 (0·17)	3·3 (0·13)	3·3 (0·12)	3·3 (0·16)
Total	11·5 (0·30)	10·8 (0·43)	11·0 (0·37)	10·5 (0·36)	10·7 (0·45)	9·7 (0·30)
**Eutrophying Emissions (g PO43-eq/g)**						
Beef	5·7 (0·27)	5·6 (0·31)	5·5 (0·30)	5·1 (0·32)	5·2 (0·41)	4·5 (0·27)
Lamb	0·7 (0·07)	0·5 (0·06)	0·6 (0·09)	0·5 (0·07)	0·6 (0·07)	0·5 (0·07)
Pork	0·6 (0·05)	0·4 (0·05)	0·4 (0·05)	0·6 (0·05)	0·6 (0·06)	0·5 (0·06)
Poultry	1·6 (0·06)	1·6 (0·12)	1·7 (0·08)	1·6 (0·06)	1·6 (0·06)	1·6 (0·08)
Total	8·6 (0·26)	8·1 (0·34)	8·2 (0·31)	7·7 (0·32)	7·9 (0·41)	7·1 (0·26)
**Freshwater Withdrawals (L/g)**						
Beef	27·5 (1·28)	27·0 (1·45)	26·4 (1·42)	24·7 (1·55)	24·9 (1·99)	21·6 (1·30)
Lamb	13·1 (1·25)	9·3 (1·07)	11·5 (1·74)	8·5 (1·34)	10·5 (1·24)	10·0 (1·26)
Pork	15·0 (1·21)	10·4 (1·12)	9·6 (1·06)	13·7 (1·24)	13·7 (1·46)	11·2 (1·34)
Poultry	21·1 (0·85)	21·6 (1·62)	23·0 (1·13)	21·4 (0·83)	21·2 (0·79)	21·4 (1·03)
Total	76·7 (2·01)	68·3 (2·57)	70·5 (2·55)	68·3 (2·27)	70·3 (2·51)	64·1 (2·04)
**Stress-Weighted Water Use (L/g)**						
Beef	658·5 (30·60)	646·7 (35·60)	630·8 (34·08)	590·4 (37·09)	595·8 (47·53)	516·0 (31·02)
Lamb	1028·3 (98·20)	728·6 (83·99)	906·7 (136·84)	669·5 (105·19)	824·1 (97·53)	785·0 (98·90)
Pork	559·9 (45·21)	386·4 (41·22)	358·7 (39·54)	510·8 (46·06)	510·4 (54·44)	417·0 (50·06)
Poultry	453·3 (18·33)	464·8 (34·87)	493·8 (24·17)	459·1 (17·93)	455·8 (16·97)	459·3 (22·04)
Total	2700·1 (108·50)	2226·5 (102·82)	2390·0 (142·05)	2229·8 (112·04)	2386·2 (110·47)	2177·3 (103·48)

Table 5. Cont. Daily environmental emissions/uses per capita by meat type in NDNS Rolling Programme (2008/09-2018/19)

	**2014/15**	**2015/16**	**2016/17**	**2017/18**	**2018/19**	**P trend**
**Land Use (m2/g)**						
Beef	5·5 (0·32)	5·1 (0·32)	5·4 (0·37)	4·7 (0·28)	4·3 (0·30)	<0·001
Lamb	1·9 (0·30)	1·6 (0·21)	1·9 (0·31)	1·7 (0·23)	1·2 (0·17)	<0·001
Pork	0·1 (0·01)	0·1 (0·01)	0·1 (0·01)	0·1 (0·01)	0·1 (0·01)	0·080
Poultry	0·5 (0·02)	0·5 (0·02)	0·5 (0·02)	0·4 (0·02)	0·4 (0·02)	0·002
Total	7·9 (0·41)	7·3 (0·33)	7·9 (0·52)	7·0 (0·34)	6·1 (0·35)	<0·001
**GHG emissions (kg CO2eq/g)**						
Beef	1·7 (0·10)	1·6 (0·10)	1·7 (0·11)	1·4 (0·09)	1·3 (0·09)	<0·001
Lamb	0·2 (0·03)	0·2 (0·02)	0·2 (0·03)	0·2 (0·03)	0·1 (0·02)	<0·001
Pork	0·1 (0·01)	0·1 (0·01)	0·1 (0·01)	0·1 (0·01)	0·1 90·01)	0·008
Poultry	0·4 (0·02)	0·4 (0·02)	0·4 (0·02)	0·4 (0·02)	0·3 (0·02)	0·002
Total	2·3 (0·10)	2·2 (0·10)	2·3 (0·13)	2·1 (0·09)	1·9 (0·09)	<0·001
**Acidifying Emissions (g SO2eq/g)**						
Beef	5·3 (0·32)	5·0 (0·31)	5·3 (0·36)	4·6 (0·28)	4·2 (0·30)	<0·001
Lamb	0·7 (0·11)	0·6 (0·08)	0·7 (0·12)	0·6 (0·09)	0·5 (0·07)	<0·001
Pork	1·0 (0·09)	1·0 (0·11)	0·9 (0·11)	0·8 (0·08)	0·8 (0·09)	0·080
Poultry	4·0 (0·19)	3·9 (0·18)	3·9 (0·19)	3·7 (0·18)	3·6 (0·20)	0·002
Total	11·0 (0·39)	10·5 (0·36)	10·8 (0·48)	9·7 (0·32)	9·1 (0·35)	<0·001
**Eutrophying Emissions (g PO43-eq/g)**						
Beef	5·1 (0·30)	4·7 (0·30)	5·0 (0·34)	4·4 (0·26)	4·0 (0·28)	<0·001
Lamb	0·5 (0·08)	0·4 (0·05)	0·5 (0·08)	0·4 (0·06)	0·3 (0·05)	<0·001
Pork	0·5 (0·05)	0·5 (0·06)	0·5 (0·06)	0·4 (0·04)	0·4 (0·05)	0·080
Poultry	1·9 (0·09)	1·9 (0·09)	1·8 (0·09)	1·7 (0·08)	1·7 (0·09)	0·002
Total	8·0 (0·32)	7·5 (0·30)	7·9 (0·39)	7·0 (0·27)	6·5 (0·29)	<0·001
**Freshwater Withdrawals (L/g)**						
Beef	24·3 (1·44)	22·7 (1·43)	24·2 (1·66)	21·1 (1·27)	19·3 (1·35)	<0·001
Lamb	9·1 (1·46)	7·8 (1·02)	9·3 (1·52)	8·3 (1·14)	6·0 (0·85)	<0·001
Pork	12·5 (1·18)	12·1 (1·34)	11·8 (1·44)	10·0 (1·02)	10·4 (1·12)	0·080
Poultry	25·7 (1·19)	25·3 (1·13)	24·9 (1·23)	23·6 (1·14)	23·3 (1·27)	0·002
Total	71·6 (2·66)	68·0 (2·29)	70·1 (3·24)	63·0 (2·10)	58·9 (2·34)	<0·001
**Stress-Weighted Water Use (L/g)**						
Beef	582·5 (34·56)	544·5 (34·32)	579·2 (39·69)	504·9 (30·31)	461·2 (32·23)	<0·001
Lamb	715·8 (114·60)	616·5 (79·92)	734·3 (119·92)	645·1 (89·76)	470·5 (66·59)	<0·001
Pork	464·4 (43·96)	451·0 (49·78)	437·72 (53·58)	373·2 (37·95)	386·8 (41·76)	0·080
Poultry	552·0 (25·63)	543·2 (24·31)	534·2 (26·46)	507·0 (24·53)	500·4 (27·34)	0·002
Total	2314·8 (129·48)	2155·2 (94·44)	2285·4 (149·80)	2039·1 (102·15)	1818·9 (94·64)	<0·001

Abbreviations: NDNS, National Diet and Nutrition Survey. Standard errors are denoted in brackets.

Figure 1. Proportion of adult consumers (≥19 years) meeting SACN's recommendation to limit red and processed meat consumption to 70g a day, n=7,243.

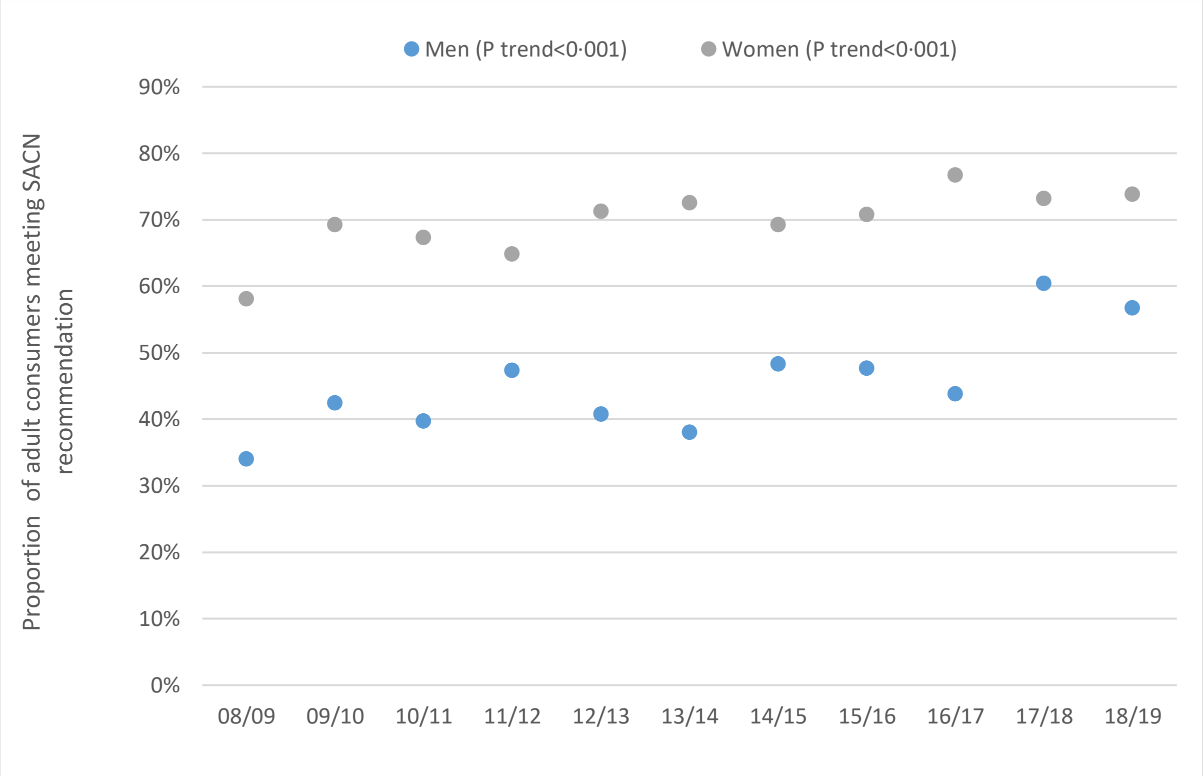

Abbreviations: SACN, Scientific Advisory Committee on Nutrition
